# Characterizing the experience of sensitive skin: A pilot survey

**DOI:** 10.1016/j.jdin.2023.02.011

**Published:** 2023-03-05

**Authors:** Erika T. McCormick, Dillon Nussbaum, Alana Sadur, Sapana Desai, Adam Friedman

**Affiliations:** Department of Dermatology, George Washington University, George Washington University School of Medicine and Health Sciences, Washington, DC

**Keywords:** cutaneous hyperactivity, patient experiences, reactive skin, SS, SS-10, Sensiscale-10, sensitive skin, sensitive skincare, sensitive skin syndrome, sensitive skin triggers, skin hyperreactivity, skin sensitivity

*To the Editor:* Sensitive skin (SS) is a subjective syndrome characterized by cutaneous hyperreactivity to otherwise innocuous stimuli.^1^ Despite being a common complaint, the pathophysiology of SS is not fully elucidated, and we do not have a complete understanding of SS. Furthermore, there are gaps in research on SS in persons of color (POC). Representative patient-reported data are essential to improve understanding of SS, given the subjectivity of this condition. We aimed to assess prevalence, symptom burden, and behavior of self-identified POC with SS.

An IRB-approved survey was completed by community health fair attendees in Washington, DC, United States after receiving brief education on SS. Compiled results were analyzed with GraphPad Prism using Fisher exact tests and Welch *t* tests (Table I). Of 58 survey respondents (89% response rate), 86% self-identified as POC. A total of 78% identified as women, and 22% identified as men. Age ranged from 18 to >75 years. At least 1 underlying skin condition was reported by 63.8% of respondents. SS was self-reported by 57% of respondents (60% of women, 46.2% of men), and 15% of them reported experiencing symptoms at least daily. A total of 27% had SS without primary skin disease. Mean scores on the Sensitive Scale-10,^2^ which is a validated assessment tool for SS severity, were significantly higher in those who reported SS than those who did not (14.61/100 vs 4.32/100, *P* = .002). Respondents with SS were more likely to report history of allergy than respondents without SS (56.25% vs 8.33%, *P* = .0002). Individuals with SS were 7 times more likely to have consulted a dermatologist (OR [95% CI], 6.86 [2.24-22.67], *P* = .0012). Of individuals with SS without primary skin disease, 80% had seen a dermatologist.

**Table I:** Study Questionnaire and Results.

		Self-reported Sensitive Skin Status	
	Response	Sensitive Skin (%)	Nonsensitive Skin (%)
Self-Reported Demographic Information			
Gender	Women	81.8	72
	Men	18.2	28
Age, y	18 to 24	15.1	2
	25 to 34	39.4	32
	35 to 44	9.1	12
	45 to 54	12.1	-
	55 to 64	6.1	16
	65 to 74	9.1	16
	≥75	6.1	4
	Prefer not to answer	3	-
Race or ethnicity *(Choose all that apply)* *No respondents selected African Caribbean, Asian American, Native American, or Alaska Native, Native Hawaiian/Other Pacific Islander.*	White/Caucasian	15.1	12
	Hispanic	9.1	12
	Black	48.5	48
	African American	36.7	20
	Multiracial	2	-
	Other	2	8
Are you employed currently?	Yes	54.5	48
	No	27.3	36
	Prefer not to answer	18.2	16
Are you a current smoker?∗	Yes	6.5	12
	No	93.5	88
Have you ever been told by a doctor that you have the following skin conditions? (*Choose all that apply*) *No respondents selected prurigo nodularis.*	Acne	45.5	24
	Rosacea	18.2	-
	Eczema/Atopic dermatitis	36.4	12
	Psoriasis	3	-
	Hives	12.1	-
	Lichen Planus	6.1	-
	Keratosis Pilaris	6.1	-
	Other	-	4
Have you had symptoms of your skin condition within the past year?∗∗	Yes	66.7	55.6
	No	33.3	44.4
Have you ever seen a dermatologist (skin doctor) before?	Yes	72.7	28
	No	27.3	72
Do you have a personal history of allergy?∗∗∗	Yes	56.25	8.33
	No	43.75	91.67
Sensitive Skin Survey Questions			
Do you think you have sensitive skin?	Yes	100	-
	No	-	100
Has anyone ever told you that you have sensitive skin?	Yes	61	4
	No	39	96
Do you have a family history of bad skin reaction to cosmetic products?∗∗∗∗	Yes	21.9	8
	No	78.1	92
Do you regularly use products for sensitive skin?∗∗∗∗	Yes	87.5	48
	No	12.5	52
Do you have skin reactions to cosmetic products or toiletries?∗∗∗∗	Yes	56.1	-
	No	43.9	100
If you have a skin reaction to cosmetic products or toiletries, is it with products that claim to be for sensitive skin? (***n = 18 who react to products)***	Yes	72	n/a
	No	28	n/a
What happens to your skin when you use products NOT for sensitive skin? (*Choose all that apply)*	Redness	33.3	n/a
	Dryness	39.4	n/a
	Itch	42.4	n/a
	Pain	15.1	n/a
	Burning	36.4	n/a
On a scale of 0-10, how bad is this reaction? *(0 = no reaction)∗∗∗∗*	*Average Score*	5.1	n/a
Does your skin have reactions to these things? (*Choose all that apply*)	Temperature change	27.3	22.4
	Extreme temperatures	48.5	16
	Wind	6.1	12
	Tap water containing limescale or metals	12.1	10.3
	Pollution	12.1	4
	Diet	36.4	16
	Skin care products	42.4	4
	Laundry detergent	33.3	8
	Hormonal changes	30.3	20
	Skin infections	3	4
	Poor hygiene	12.1	4
	Medication	12.1	4
	Stress	39.4	28
	Sweat	39.4	24
	Sun exposure	36.4	20
	Chlorine	18.2	15.5
	Alcohol	12.1	8
	Other	3	-
	None of the above	6.1	44
What happens to your skin when it reacts to these things? *(Choose all that apply)*	Redness	45.5	24
	Dryness	60.6	44
	Itch	45.5	28
	Pain	6.1	8
	Burning	24.2	16
How often do you suffer from sensitive skin (discomfort of skin like tightness, itching, stinging, burning, or pain)?	> Once a day	6.1	-
	Once a day	9.1	-
	Few times a week	12.1	4
	Every few weeks	18.2	8
	Every few months	6.1	-
	Few times a year	27.2	24
	Less frequently	12.1	40
	*No answer*	9.1	24
Sensitive Scale-10 Score, *mean (out of 100)*			

How irritated has your skin been during the past 3 days? Choose a number on a scale of 0-10, where 0 = not irritated/normal. Have you had any of these skin symptoms during the past 3 days? Choose a number from 0-10 to describe the severity of each symptom, where 0 = no symptom. • Tingling • Burning • Sensations of heat • Tightness/tautness • Itching • Pain • General Discomfort • Hot flashes • Redness		14.61	4.32

Percentages were calculated using the number of respondents who answered each question; n = 33 for sensitive skin (SS), and n = 25 for nonsensitive skin (non-SS), unless stated otherwise: ∗SS n = 31; ∗∗SS n = 24, non-SS n = 9; ∗∗∗SS n = 32, Non-SS n = 24; ∗∗∗∗SS n = 32.

Hyperreactivity to consumer products was reported by 56% of individuals with SS, which manifested as cutaneous symptoms of burning (56%), itch (56%), redness (39%), dryness (39%), and pain (17%). Mean severity score of these reactions from 0 to 10 (0 = no reaction) was 5.1 (SD, 2.14). Respondents with SS were 7.5 times more likely to use products marketed for SS (OR, 7.58 [1.99-23.80], *P* = .0028), and notably, 72% of those with hyperreactivity also reported reactions to SS products. One or more triggers causing skin reactions were reported by 94% of respondents with SS (Fig 1). The average number of triggers per individual with SS was 4.2 (SD, 3.54), which was significantly more than respondents without SS (*p* =.0128). The most commonly reported SS triggers included extreme temperatures (48%), skincare products (42%), stress (39%), sweat (39%), sun exposure (36%), and diet (36%); 91% of individuals with SS reacted to at least 1 of these.

**Fig 1:**
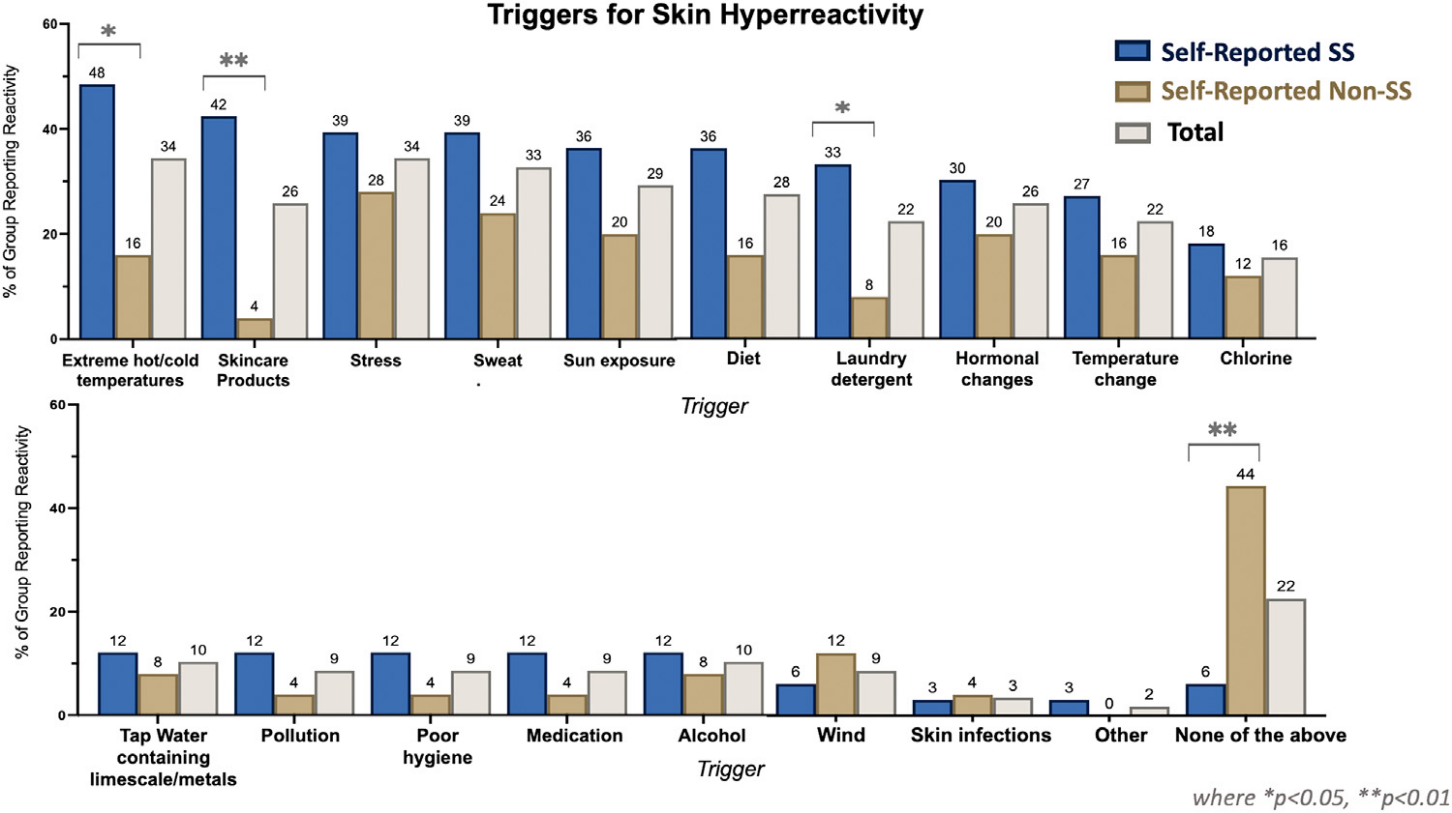
Triggers for Skin Hyperreactivity. 58 survey respondents (33 with self-reported sensitive skin, 25 with self-reported nonsensitive skin) were instructed to choose all triggers that caused a skin reaction for them.

These data contribute to an improved understanding of the subjective experience of SS. SS is a common phenomenon that impacted over half of the individuals surveyed. Despite a relatively small sample size, our findings were consistent with large epidemiological studies.^1^, ^3^, ^4^ This work identified pitfalls, such as high rates of reactivity to products marketed for SS, and potential therapeutic/management targets, such as cholinergic stimuli, to offer better approaches to managing SS. Although this is the first survey in a predominant POC population within the United States, further research is required to validate the patterns observed in this study. Future directions include reconducting this pilot survey among a larger population.

## Conflicts of interest

Ms McCormick’s research is funded by the Galderma Sensitive Skin Fellowship Program.
